# Comparison of the nutrient composition of eggs produced in the Guatemalan highlands during the wet and dry seasons

**DOI:** 10.1002/fsn3.3736

**Published:** 2023-10-13

**Authors:** Taylor C. Wallace, Gabriela Montenegro‐Bethancourt, Peter Rohloff, Elizabeth Yakes Jimenez, Gabriela V. Proaño, George P. McCabe, Alison Steiber, Andrew Ruosch, Ian Laessig, Edward Ladwig, Hong You

**Affiliations:** ^1^ Think Healthy Group, LLC Washington District of Columbia USA; ^2^ School of Medicine and Health Sciences George Washington University Washington District of Columbia USA; ^3^ Gerald J. and Dorothy R. Friedman School of Nutrition Science and Policy Tufts University Medford Massachusetts USA; ^4^ Academy of Nutrition and Dietetics Chicago Illinois USA; ^5^ Wuqu' Kawoq/Maya Health Alliance Tecpan Guatemala; ^6^ Brigham and Women's Hospital Boston Massachusetts USA; ^7^ College of Population Health and Department of Pediatrics and Internal Medicine University of New Mexico Health Sciences Center Albuquerque New Mexico USA; ^8^ Department of Statistics Purdue University West Lafayette Indiana USA; ^9^ Department of Nutrition Case Western University Cleveland Ohio USA; ^10^ Eurofins Food Chemistry Testing Madison, Inc. Madison Wisconsin USA; ^11^ Eurofins U.S. Food Des Moines Iowa USA; ^12^ Eurofins Botanical Testing US, Inc. Brea California USA

**Keywords:** chickens, choline, eggs, food analysis, nutritional status, ovum

## Abstract

The potential of chicken eggs as a nutritionally complete protein and source of key micronutrients during the first 1000 days post‐conception has been progressively recognized across the globe, particularly in resource‐poor settings. Fluctuation of egg nutrient content by season is relatively unknown, which may influence international food composition databases and outcomes in intervention studies using egg supplementation. To better interpret the findings of The Saqmolo' Project, we conducted comprehensive nutrient analyses on eggs produced during the wet and dry seasons in the highlands of central Guatemala. We randomly collected 36 shell eggs from a local farm during both seasons, hard‐boiled, and prepared them for transport to the United States, where they were pooled and assessed for their nutrient composition. Methods of the Association of Official Analytical Chemists, the American Oil Chemists Society, and the American Association of Cereal Chemists were utilized to determine total energy, moisture, ash, total protein, total fat, fatty acids, total carbohydrates, 12 vitamins, 11 minerals, and carotenoids, by season, in some instances with modifications. Differences in nutrient composition between de‐shelled hard‐boiled eggs collected between seasons were assessed using an analysis of variance (ANOVA) and Tukey's family error rate comparison test. Most nutrients in eggs produced in the highlands of central Guatemala differed negligibly (but statistically significantly) based on seasonality. Only vitamins A and E, folate, choline, and calcium fluctuated at clinically significant levels relative to the AI/RDA for infants 7–12 months. Total energy, protein, trans fatty acids, moisture, and vitamin D3 levels did not differ between seasons (*p* > .05). Further multi‐year sampling is needed to examine how seasonal variation affects the nutrient composition of eggs. These data may be used to supplement existing national and regional food composition databases.

## Graphical abstract

To better interpret the findings of The Saqmolo’ Project, we conducted comprehensive energy, moisture, ash, total protein, total fat, fatty acids (monounsaturated: MUFA, polyunsaturated: PUFA, saturated, unsaturated, omega‐3, omega‐6, omega‐9, and individual fatty acids), total carbohydrates, dietary fiber, vitamins (biotin, choline, folate, niacin, pantothenic acid, riboflavin, thiamin, vitamins A, B6, B12, D, and E, minerals (calcium, copper, iodine, iron, magnesium, manganese, phosphorus, potassium, selenium, sodium, and zinc), and carotenoids (lutein and zeaxanthin) analyses on eggs produced during the wet and dry seasons in the highlands of central Guatemala.

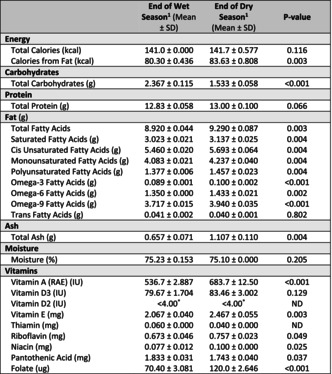

## 1 INTRODUCTION

The potential of chicken eggs as a nutritionally complete protein and source of key micronutrients has been progressively recognized across the globe, particularly in resource‐poor settings (Iannotti et al., 2014). Eggs have a high nutrient‐to‐energy density and contain a wide array of complete proteins, fats, vitamins, and minerals (Iannotti et al., 2014). They are also among the more affordable and environmentally sustainable animal‐sourced foods (Drewnowski, 2010; Walker & Baum, 2022). Eggs have been identified as the lowest‐cost animal‐sourced food for protein, vitamin A, iron, vitamin B12, riboflavin, and choline and the second‐lowest‐cost source for zinc and calcium (Drewnowski, 2010). The nutritional composition of chicken eggs varies partially on the breed (Ricklefs, 1977; Sinanoglou et al., 2011; Sotherland & Rahn, 1987). Egg nutrient content is also influenced by hen age, as well as environmental factors such as drought, rainfall, housing systems, and feed composition (Antova et al., 2019). In whole, raw eggs, water, protein, fat, carbohydrates, and ash comprise about 76.1%, 12.6%, 9.5%, 0.7%, and 1.1% of the egg, respectively (Hayowitz et al., 2022). The composition of the yolk and white differ substantially in regard to their nutrient content. The content of most nutrients in eggs is largely dependent on the ratio of egg white to yolk, as the majority reside in the yolk. Total protein and water content of egg white varies from 9.9% to 10.7% and from 46.8% to 88.3%, respectively (Mróz et al., 2014). The egg yolk contains a relatively complex array of lipids and lipoproteins that constitute about one‐third of its composition (“Lipids,” 2009). This lipid matrix consists mainly of triglycerides (62.3%), phospholipids (32.8%), and sterols (cholesterol 5.0%). Egg lipids are high in monosaturated fatty acid (MUFA) and unsaturated fatty acid contents (de Koning, 1974; “Lipids,” 2009). The protein and water content of the yolk varies from 16.2% to 17.9% and 45.9% to 46.8%, respectively (Mróz et al., 2014). Egg yolks are the primary source of choline in the Western diet (HHS & USDA, 2015). Phosphatidylcholine is the major choline‐containing phospholipid constituent (45%–60%) present, followed by phosphatidylethanolamine, phosphatidylinositol, and lysophosphatidylcholine. Lysophosphatidylcholine, lysophosphatidylethanolamine, and sphingomyelin are other common egg yolk components (Antova et al., 2019). Polyunsaturated fatty acids (PUFAs) in the main phospholipid fraction are 30%–40%, saturated fatty acids are 45%, and MUFAs are 20%–25%. The phospholipids (mainly phosphatidylcholine) in the egg yolk also contain docosahexaenoic acid (DHA), which like choline is more bioavailable in this form (Carnielli et al., 1998; Cook et al., 2016; Maki et al., 2009; Ramprasath et al., 2013, 2015; Smolders et al., 2019; Ulven et al., 2011). Egg yolks are also a rich source of the bioactive xanthophyll carotenoid, lutein, which has been shown to exhibit much higher bioavailability in this matrix as compared to spinach and lutein ester supplements (Chung et al., 2004; Handelman et al., 1999). The fatty acid profile of the egg yolk and content of micronutrients and carotenoids can differ substantially with varying hen diets. The content of some minerals (e.g., selenium, iodine, iron, zinc, and magnesium) and fat‐ and water‐soluble vitamins can be easily enhanced by increasing the hen's dietary intake of these nutrients (Nys et al., 2011; Réhault‐Godbert et al., 2019). As such, because birds in free‐rearing systems have access to grains, insects, and worms in addition to their basic diet, certain nutrients in eggs may vary (Nys et al., 2011).

The highlands of central Guatemala are home to historically marginalized and exploited Indigenous Maya populations. In 2015, the prevalence of stunting (i.e., length‐for‐age [LAZ] z‐core <2.0) among children under the age of 2‐years was estimated to be approximately 42.4% (Global Nutrition Report, 2022). The diets of Guatemalan children, particularly Indigenous Maya children, have been shown to lack dietary diversity and animal‐sourced foods (Webb et al., 2016). Eggs contribute clinically relevant quantities of all 14 nutrients (except glucose) considered key for supporting brain development during the first 1000 days of life (Iannotti et al., 2014; Ranganathan et al., 2016; Schwarzenberg et al., 2018). However, the nutritional composition of eggs in the highlands of Guatemala could vary due to dramatic weather shifts between seasons. The seasons in Guatemala used to be predictable, with a dry season prevailing November through April and a wet season May through October (Britannica, 2022; Pionero Philanthropy, 2021). Severe tropical storms traditionally climax during the wet months of September and October; however due to climate change, these seasons are becoming more erratic and less predictable (Britannica, 2022; Pionero Philanthropy, 2021). Prolonged droughts and floods can have a large impact on agriculture production and animal feed supply chains. These potential changes have largely been undocumented in food composition databases.

The objective of this study was to evaluate the nutrient composition of eggs utilized in The Saqmolo’ Project during the wet and dry seasons in the highlands of Guatemala. The Saqmolo’ (i.e., “egg” in the Mayan language, Kaqchiquel) Project is an individually randomized, partially blinded, controlled comparative effectiveness trial to evaluate the influence of adding delivery of a single whole egg per day to local standard nutrition care (i.e., growth monitoring, medical care, deworming medication, multiple micronutrient powders for point‐of‐use food fortification, and individualized complementary and responsive feeding education for caregivers) for 6 months, compared with the local standard nutrition care package alone, on child development, growth, and diet quality measures in rural indigenous Maya infants aged 6–9 months at baseline (*N* = 1200). The study is being implemented by Wuqu’ Kawoq – Maya Health Alliance (MHA) and the protocol has been published (Wallace et al., 2022).

## 2 METHODS

### 2.1 Sample collection, preparation, transport, and storage

We followed previously published procedures for sample collection, preparation, transport, and storage used in assessing the nutrient composition of eggs from a similar clinical trial conducted in Malawi (Caswell et al., 2021; Werner et al., 2019). On two separate time points, 9 November 2021 (end of wet season) and 29 March 2022 (end of dry season), a total of 36 fresh shell intact eggs (mean 65.2 ± 3.84 g; range 57.00–70.88 g) produced the same day from a local commercial chicken egg farm production facility about 30 km distance from Tecpán, Guatemala, were selected at random from a pallet containing thousands of eggs of the same size grading and transported to the MHA headquarters (~15 min by car). The production facility is operated by an independent commercial producer using its own farm management practices and sanitary standard operating procedures established by the Guatemalan government. Commercial Lohmann brown and white chickens produced the eggs for The Saqmolo' Project. We were unable to obtain the formulation of the feed used; however, production facility staff were able to confirm that feed was consistent between the sample collection timepoints (i.e., same supplier, brand, and SKU). Upon arrival to MHA headquarters, eggs were immediately weighed with the shell intact using a Uline Balance Scale – 220 g × 0.01 g (Pleasant Prairie, WI), hard‐boiled for 4 min, placed in plastic cartons, sealed, and stored on ice for same‐day transportation to Washington, DC, USA. Upon arrival, hard‐boiled eggs were stored overnight in a conventional refrigerator at 38°F (276.5 K). The following morning, the hard‐boiled eggs were encased in ice packs and shipped from Washington, DC same day to the Eurofins lab in Madison, Wisconsin, where they were further refrigerated at or below 38°F (276.5 K). Damaged eggs (*n* = 3, end of wet season; *n* = 2, end of dry season) were discarded prior to storage in the refrigerator at 38 °F (276.5 K) for a maximum of 5 days prior to sample preparation. Egg yolk total lipids, fatty acid composition, Haugh units, and tocopherol content of hard‐boiled eggs have previously been shown not to differ within 1 week of storage at refrigeration temperature (Ahn et al., 1995). Shells of the undamaged hard‐boiled eggs were removed and discarded, and the whites and yolks were homogenized and pooled separately by seasonal batch (*n* = 33, end of wet season; *n* = 34, end of dry season) for the various nutrient analyses described below. We had previously estimated that homogenizing a minimum of 18 whole hard‐boiled eggs (~1200 g) per seasonal batch would be sufficient for conducting all intended nutrient analyses. Pooling of the large number of eggs within each seasonal batch enabled us to provide measures that reflect the average nutrient content of a large number of samples at a reasonable cost.

### 2.2 Analytical testing

Analysis of pooled seasonal batch samples were conducted in a laboratory accredited to ISO/IEC 17025 standards of the International Organization for Standardization/International Electrotechnical Commission (ISO/EC) for most nutrient analyses. In general, the laboratory utilized official methods of the Association of Official Analytical Chemists (AOAC), the American Oil Chemists Society (AOCS), and the American Association of Cereal Chemists (AACC) outlined, in some instances with modifications, for specific nutrient analysis as presented in Table 1. The present study reports energy, moisture, ash, total protein, total fat, total fatty acids (monounsaturated: MUFA, polyunsaturated: PUFA, saturated, unsaturated, omega‐3, omega‐6, omega‐9, and individual fatty acids), total carbohydrates, 12 vitamins (biotin, choline, folate, niacin, pantothenic acid, riboflavin, thiamin, vitamins A, B_6_, B_12_, D, and E), 11 minerals (calcium, copper, iodine, iron, magnesium, manganese, phosphorus, potassium, selenium, sodium, and zinc), and two carotenoids (lutein and zeaxanthin) per 100 g of sample. Each analytical test was conducted in triplicate for each seasonal batch of pooled samples to ensure precision of the laboratory estimates.

**TABLE 1:** Methods used for nutrient analyses of eggs.

Component	Method reference^a^
Ash	AOAC 923.03. Ash of flour. [Direct method]
Biotin	Procedures of the SEB. Determination of biotin with *Lactobacillus arabinosis* (modified). [Microbiological assay]. (Scheiner & De Ritter, 1975; Wright & Skegs, 1944)
Carbohydrates	USDA. Energy value of foods. Agriculture handbook No 74. (Merrill & Watt, 1973)
Carotenoids	AOAC 941.15. Carotene in fresh plant materials and silages. [Spectrophotometric method] (Forrest, 1987)
Choline	AOAC 2015.10. Free and total choline and carnitine in infant formula and adult/pediatric nutritional formula. [Liquid chromatography/tandem mass spectrometry]
Energy	USDA. Energy value of foods. Agriculture handbook No 74. (Merrill & Watt, 1973)
Fatty acids	AOAC 996.06. Fat (total, saturated, and unsaturated) in foods. [Gas chromatography]
Folate	AOAC 960.46. Vitamin assays. [Microbiological assay]
Iodine	AOAC 2012.15. [ICP emission spectrometry]
Minerals (Ca, Cu, Fe, Mg, Mn, P, K, Na, Zn)	AOAC 984.27, 985.01, and 2011.14. Metals and other elements in plants and pet foods. [ICP emission spectrometry]
Moisture	AOAC 925.09. Solids (total) and moisture in flour. [Gravimetry, vacuum oven]
Niacin	AOAC 944.13 and 960.46. Vitamin assays. [Microbiological assay]
Pantothenic acid	AOAC 945.74, 992.07, and 960.46. Vitamin assays. [Microbiological assay]
Protein	AOAC 968.06 and 992.15. Protein (crude) in animal feed. [Dumas method]
Riboflavin	AOAC 940.33 and 960.46. Vitamin assays. [Microbiological assay]
Selenium	AOAC 2011.19. Chromium, selenium, and molybdenum in infant formula and adult nutritional products. [Inductively coupled plasma‐mass spectrometry]
Thiamin	AOAC 942.23, 953.17, and 957.17. Thiamin (vitamin B_1_). [Fluorometric method]
Vitamin A	AOAC 992.04, 992.06, and 2001.13. Vitamin A (retinol) in foods. [Liquid chromatography]
Vitamin B6	AOAC 961.15. Vitamin B_6_ (pyridoxine, pyridoxal, pyridoxamine) in food extracts. [Microbiological method]
Vitamin B12	AOAC 952.20 and 960.46. Vitamin assays. [Microbiological assay]
Vitamin D	AOAC 2011.11. Vitamin D in infant formula and adult/pediatric nutritional formula. [Ultra‐high‐performance liquid chromatography/tandem mass spectrometry] (Huang et al., 2009)
Vitamin E	AACC Method 86‐06.01. Analysis of vitamins A and E (modified). [High‐performance liquid chromatography fluorescence]. (Cort et al., 1983; McMurray et al., 1980; Speek et al., 2006)

Abbreviations: AOAC, Association of Official Analytical Chemists; AOCS, American Oil Chemists Society; AACC, American Association of Cereal Chemists.

^a^ Methods in use by accredited commercial laboratory in 2022. Additional references cited. Details on individual methods used are available from the authors and Eurofins US Food.

### 2.3 Statistical analyses

Means and standard deviations were calculated for each of the selected nutrients. The difference in nutrient composition in eggs collected between seasons in Guatemala was assessed using an analysis of variance (ANOVA) and Tukey's family error rate comparison test. Differences were considered significant when *p* ≤ .05. All statistical analyses were carried out using Microsoft Excel for Mac, version 16.66 (Microsoft Corporation; Redmond, WA USA).

## 3 RESULTS

Eggs collected and analyzed in our study weighed 65.2 ± 3.84 g (range 57.00–70.88 g). Energy, moisture, ash, total protein, total fat, fatty acids, total carbohydrates, 12 vitamins, 11 minerals, and carotenoids of eggs collected during the end of the wet and end of the dry seasons in the highlands of central Guatemala are presented in Table 2.

**TABLE 2:** Nutrient composition of the edible portion of de‐shelled hard‐boiled eggs collected during the end of the wet and dry seasons (expressed as per 100 g fresh weight).

	End of wet season^b^ (mean ± SD)	End of dry season^b^ (mean ± SD)	*p*‐Value
Energy			
Total calories (kcal)	141.0 ± 0.000	141.7 ± 0.577	.116
Calories from fat (kcal)	80.30 ± 0.436	83.63 ± 0.808	.003
Carbohydrates			
Total carbohydrates (g)	2.367 ± 0.115	1.533 ± 0.058	<.001
Protein			
Total protein (g)	12.83 ± 0.058	13.00 ± 0.100	.066
Fat (g)			
Total fatty acids	8.920 ± 0.044	9.290 ± 0.087	.003
Saturated fatty acids (g)	3.023 ± 0.021	3.137 ± 0.025	.004
Cis unsaturated fatty acids (g)	5.460 ± 0.020	5.693 ± 0.064	.004
Monounsaturated fatty acids (g)	4.083 ± 0.021	4.237 ± 0.040	.004
Polyunsaturated fatty acids (g)	1.377 ± 0.006	1.457 ± 0.023	.004
Omega‐3 fatty acids (g)	0.089 ± 0.001	0.100 ± 0.002	<.001
Omega‐6 fatty acids (g)	1.350 ± 0.000	1.433 ± 0.021	.002
Omega‐9 fatty acids (g)	3.717 ± 0.015	3.940 ± 0.035	<.001
Trans fatty acids (g)	0.041 ± 0.002	0.040 ± 0.001	.802
Ash			
Total ash (g)	0.657 ± 0.071	1.107 ± 0.110	.004
Moisture			
Moisture (%)	75.23 ± 0.153	75.10 ± 0.000	.205
Vitamins			
Vitamin A (RAE) (IU)	536.7 ± 2.887	683.7 ± 12.50	<.001
Vitamin D3 (IU)	79.67 ± 1.704	83.46 ± 3.002	.129
Vitamin D2 (IU)	<4.00^a^	<4.00^a^	ND
Vitamin E (mg)	2.067 ± 0.040	2.467 ± 0.055	.003
Thiamin (mg)	0.060 ± 0.000	0.040 ± 0.000	ND
Riboflavin (mg)	0.673 ± 0.046	0.757 ± 0.023	.049
Niacin (mg)	0.077 ± 0.012	0.100 ± 0.000	.025
Pantothenic acid (mg)	1.833 ± 0.031	1.743 ± 0.040	.037
Folate (μg)	70.40 ± 3.081	120.0 ± 2.646	<.001
Vitamin B6 (μg)	0.127 ± 0.005	0.153 ± 0.003	.001
Vitamin B12 (μg)	1.690 ± 0.010	1.450 ± 0.104	.016
Biotin (μg)	30.30 ± 1.200	26.20 ± 0.173	.004
Choline (mg)	321.3 ± 5.508	388.0 ± 5.568	<.001
Minerals			
Calcium (mg)	45.67 ± 0.551	111.7 ± 4.509	<.001
Copper (mg)	0.063 ± 0.002	0.074 ± 0.006	.050
Iron (mg)	1.710 ± 0.017	2.013 ± 0.015	<.001
Magnesium (mg)	10.67 ± 0.058	11.53 ± 0.058	<.001
Manganese (mg)	0.023 ± 0.00	0.030 ± 0.001	<.001
Phosphorus (mg)	183.3 ± 1.528	190.7 ± 0.577	.002
Potassium (mg)	121.0 ± 1.000	130.7 ± 0.577	<.001
Sodium (mg)	139.7 ± 1.528	136.3 ± 0.577	.024
Zinc (mg)	1.107 ± 0.012	1.270 ± 0.017	<.001
Iodine (μg)	37.87 ± 0.681	39.37 ± 0.153	.020
Selenium (μg)	24.33 ± 0.153	27.00 ± 0.529	.001
Carotenoids			
Lutein (μg)	3.490 ± 0.072	3.207 ± 0.284	.170
Zeaxanthin (μg)	1.870 ± 0.044	2.207 ± 0.226	.064

Abbreviation: ND, not determined due to lack of variance between samples.

^a^ Below lower limit of detection.

^b^ A total of 33 (end of dry season batch) and 34 (end of wet season batch) undamaged de‐shelled eggs were pooled and homogenized for the nutrient analyses. Each analytical test was conducted in triplicate for each pooled sample.

Most nutrients showed negligible (but statistically significant) differences between measurements relative to the AI/RDA for infants 7–12 months. Total energy, protein, trans fatty acids, moisture, vitamin D_3_, lutein, and zeaxanthin levels did not differ between season (*p* > .05). Calories from fat and ash were negligibly higher in eggs collected during the end of the dry season, whereas carbohydrates were negligibly higher during the end of the wet season (*p* < .05).

Eggs collected at the end of the dry season contained more total fat, saturated fatty acids, cis‐unsaturated fatty acids, monounsaturated fatty acids (MUFA), polyunsaturated fatty acids (PUFA), omega‐3 fatty acids, omega‐6 fatty acids, and omega‐9 fatty acids, as compared to samples collected at the end of the wet season (*p* < .05), although differences were small (Table 2). Palmitoleic acid differed from other individual fatty acids tested, in that it was negligibly higher during the end of the wet season (*p* < .05). All other fatty acids were slightly higher in eggs collected at the end of the dry season (*p* < .05), aside from myristic, homogamma linolenic, and docosapentaenoic acids, whose difference could not be determined due to lack of variance, or the amount was below the instrument detection limit (Table 3).

**TABLE 3:** Fatty acid composition of the edible portion of de‐shelled hard‐boiled eggs collected during the end of the wet and dry seasons (expressed as per 100 g fresh weight excluding the shell).

	End of wet season^b^ (mean ± SD)	End of dry season^b^ (mean ± SD)	*p*‐Value
Saturated fatty acids			
4:0 Butyric (g)	<0.007^a^	<0.01^a^	–
6:0 Caproic (g)	<0.007^a^	<0.01^a^	–
8:0 Caprylic (g)	<0.007^a^	<0.01^a^	–
10:0 Capric (g)	<0.007^a^	<0.01^a^	–
12:0 Lauric (g)	<0.007^a^	<0.01^a^	–
14:0 Myristic (g)	0.034 ± 0.00	0.032 ± 0.00	ND
15:0 Pentadecanoic (g)	<0.007^a^	<0.01^a^	–
16:0 Palmitic (g)	2.387 ± 0.015	2.443 ± 0.021	.019
17:0 Heptadecanoic (g)	0.011 ± 0.001	0.014 ± 0.001	.003
18:0 Stearic (g)	0.741 ± 0.006	0.798 ± 0.006	<.001
20:0 Arachidic (g)	<0.007^a^	<0.01^a^	–
22:0 Behenic (g)	<0.007^a^	<0.01^a^	–
24:0 Lignoceric (g)	<0.007^a^	<0.01^a^	–
Monounsaturated fatty acids			
14:1 Myristoleic (g)	<0.007^a^	<0.01^a^	–
15:1 Pentadecenoic (g)	<0.007^a^	<0.01^a^	–
16:1 Palmitoleic (g)	0.333 ± 0.002	0.287 ± 0.003	<.001
17:1 Heptadecenoic (g)	<0.007^a^	<0.01^a^	–
18:1 Oleic (g)	3.697 ± 0.015	3.920 ± 0.035	<.001
20:1 Eicosenoic (g)	0.023 ± 0.00	0.024 ± 0.001	.116
22:1 Erucic (g)	<0.007^a^	<0.01^a^	–
24:1 Nervonic (g)	<0.007^a^	<0.01^a^	–
Polyunsaturated fatty acids			
18:2 Linoleic (g)	1.070 ± 0.000	1.153 ± 0.012	<.001
18:3 Gamma Linolenic (g)	<0.007^a^	0.010 ± 0.001	–
18:3 Alpha Linolenic (g)	0.029 ± 0.000	0.030 ± 0.001	.016
18:4 Octadecatetraenoic (g)	<0.007^a^	<0.01^a^	–
20:2 Eicosadienoic (g)	0.012 ± 0.000	0.012 ± 0.000	ND
20:3 Eicosatrienoic (*n3*) (g)	<0.007^a^	<0.01^a^	–
20:3 Homogamma Linolenic (*n6*) (g)	0.016 ± 0.000	0.014 ± 0.000	ND
20:4 Arachidonic (*n3*) (g)	<0.007^a^	<0.01^a^	–
20:4 Arachidonic (*n6*) (g)	0.161 ± 0.002	0.171 ± 0.003	.005
20:5 Eicosapentaenoic (g)	<0.007^a^	<0.01^a^	–
21:5 Heneicosapentaenoic (g)	<0.007^a^	<0.01^a^	–
22:2 Docosadienoic (g)	<0.007^a^	<0.01^a^	–
22:3 Docosatrienoic (g)	<0.007^a^	<0.01^a^	–
22:4 Docosatetraenoic (g)	0.018 ± 0.001	0.020 ± 0.000	.002
22:5 Docosapentaenoic (*n3*) (g)	0.008 ± 0.00	0.010 ± 0.000	ND
22:5 Docosapentaenoic (*n6*) (g)	0.059 ± 0.000	0.058 ± 0.004	.598
22:6 Docosahexaenoic (*n3*) (g)	0.052 ± 0.001	0.060 ± 0.002	.002

Abbreviation: ND, not determined due to lack of variance between samples.

^a^ Below lower limit of detection.

^b^ A total of 33 (end of dry season batch) and 34 (end of wet season batch) undamaged de‐shelled eggs were pooled and homogenized for the nutrient analyses. Each analytical test was conducted in triplicate for each pooled sample.

Vitamins A, B_6_, and E, niacin, riboflavin, folate, and choline levels were higher in eggs collected at the end of the dry season, while thiamin, pantothenic acid, vitamin B_12_, and biotin levels were higher in eggs collected at the end of the wet season (*p* < .05). Likewise, calcium, copper, iron, magnesium, manganese, phosphorus, potassium, zinc, iodine, and selenium levels were higher in eggs collected during the end of the dry season, while only sodium was higher in eggs collected at the end of the wet season (*p* < .05).

**FIGURE 1:**
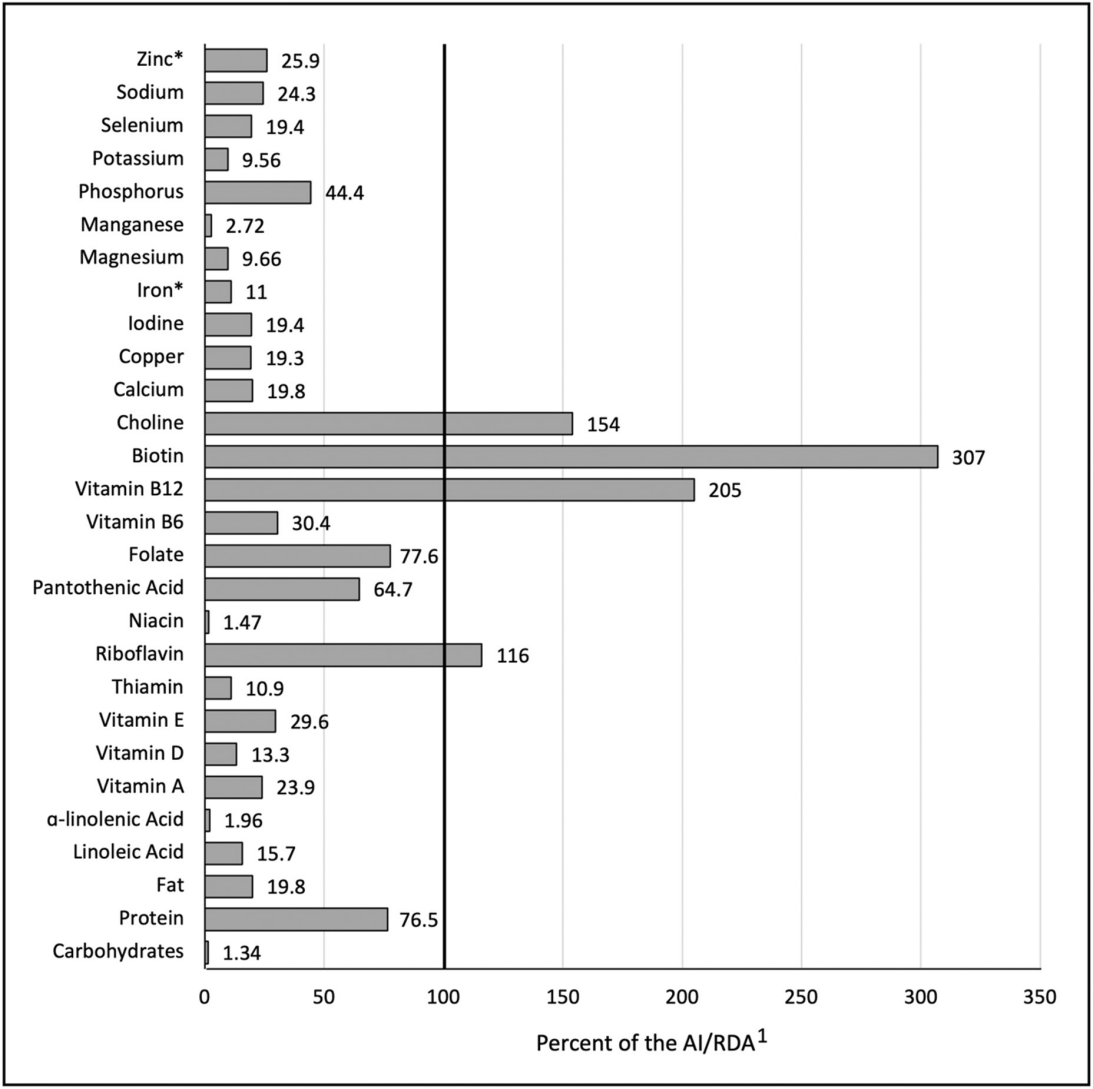
Estimated percentage (nutrient content) of a typical de‐shelled, hard‐boiled egg used in The Saqmolo' Project in relation to the current AI or RDA for infants aged 7–12 months. ^1^Dietary Reference Intakes (DRIs) published by the National Academy of Medicine (Committee to Review the Dietary Reference Intakes for Sodium and Potassium et al., 2019; Institute of Medicine (U.S.), 2001; Institute of Medicine (US) Panel on Dietary Antioxidants and Related Compounds, 2000; Institute of Medicine (US) Standing Committee on the Scientific Evaluation of Dietary Reference Intakes, 1997; IOM, 1998; Otten et al., 2006; Ross & Institute of Medicine (U. S.), 2011). *Denotes Recommended Dietary Allowance (RDA). AI, Adequate Intake; RDA, Recommended Dietary Allowance.

On average, we estimate that eggs used in The Saqmolo’ Project provided >100% of the defined AI for biotin, choline, vitamin B_12_, and riboflavin, >75% of the AI for protein and folate, >50% of the AI for pantothenic acid, and >25% of the AI/RDA for vitamin B_6_, vitamin E, phosphorus, and zinc (Figure 1).

## 4 DISCUSSION

The nutritional composition of eggs obtained in the highlands of Guatemala remained relatively consistent between seasons, with only vitamins A and E, folate, choline, and calcium showing fluctuations that would be considered significant from a clinical standpoint in infants 7 to 12 months of age. These differences could be consequential over time in a resource‐poor environment where dietary diversity is suboptimal.

Nutrient composition of eggs has been shown to differ based on a number of genetic and environmental factors, including seasonality (Réhault‐Godbert et al., 2019). Hen nutrition is also crucial for optimizing egg quality (Bouvarel et al., 2011; Réhault‐Godbert et al., 2019). The egg profile in fatty acids and micronutrients such as vitamins A, D, E, and K, iodine, selenium, and manganese has been shown to directly reflect the supply of these nutrients (Bouvarel et al., 2011; Nys et al., 2011). Yolk color also depends on the dietary supply of carotenoids (Bouvarel et al., 2011). Although the farm was unable to provide the formulation of the feed used, they were able to confirm that it was consistent between the two sample collection dates. It is possible that the nutrient composition of the commercial feed provided to the hens could fluctuate, depending on environmental growing conditions, storage time, and sourcing. In addition, since birds in free‐rearing systems have access to auxiliary grains, insects, and worms in addition to their commercial diet, seasonality may also indirectly influence hen dietary intake (Nys et al., 2011). For example, hens may have less access to grazing during the wet season due to heavy rainfall.

Others have argued that eggs are uniquely positioned to support early childhood growth and development, especially in vulnerable populations living in resource‐poor environments (Iannotti et al., 2014). The American Academy of Pediatrics has the position that although all nutrients are necessary for brain development during the first 1000 days post‐conception, key nutrients that support neurodevelopment include protein, long‐chain fatty acids, glucose, vitamins A, K, B_6_, B_12_, folate, choline, zinc, copper, iodine, iron, and selenium (Schwarzenberg et al., 2018). The first 1000 days is a nutrition‐sensitive time period, after which developmental deficits may result, even despite subsequent nutrient repletion (Schwarzenberg et al., 2018). Indeed, the eggs in our study provided substantial amounts of most nutrients considered to be key for brain development by AAP, relative to the current AIs (or RDAs in the case of zinc and iron) defined by the U.S. National Academy of Medicine (Committee to Review the Dietary Reference Intakes for Sodium and Potassium et al., 2019; Institute of Medicine (U.S.), 2001; Institute of Medicine (US) Panel on Dietary Antioxidants and Related Compounds, 2000; Institute of Medicine (US) Standing Committee on the Scientific Evaluation of Dietary Reference Intakes, 1997). Exceptions include vitamin K and glucose, which were not measured in our study but have been reported in standard reference databases to be present in only negligible amounts in eggs (U.S. Department of Agriculture, Agriculture Research Service, 2022; Instituto de Nutrición de Centro América y Panamá, 2012).

Eggs in this study were more nutrient dense compared to what has been reported in the U.S. Department of Agriculture's FoodData Central (record number 173424) and the Instituto de Nutrición de Centro América y Panamá food composition tables (U.S. Department of Agriculture, Agriculture Research Service, 2022; Instituto de Nutrición de Centro América y Panamá, 2012). However, the nutritional composition of eggs in our study did not largely differ from the composition reported in a comparable clinical study conducted in Malawi that utilized similar (note: longer transport time from Africa to the U.S.) collection, preparation, transport, and storage procedures (Caswell et al., 2021; Werner et al., 2019). Eggs in our study contained substantial amounts of omega‐3 fatty acids (e.g., DHA) and a much higher ratio of linoleic acid (18:2, n‐6) to ɑ‐linolenic acid (18:3, n‐3) than what is needed for efficient conversion of ɑ‐linolenic acid to DHA (Institute of Medicine (U.S.) & Institute of Medicine (U.S.), 2005). The choline content of our eggs in both seasons (321 and 388 mg/100 g) was higher than in eggs collected in Malawi (238 mg/100 g) and levels reported in the U.S. Department of Agriculture's Food Data Central (294 mg/100 g). Choline and DHA have been thought to have a functional synergistic relationship in infant neurocognitive development, as recently reviewed by experts in the field (Mun et al., 2019). Aside from choline, the nutritional composition of eggs in our study were also relatively similar with other investigations, as recently summarized by others (Réhault‐Godbert et al., 2019).

Our study has several strengths and limitations. The major strengths of our study include the analysis of eggs produced during multiple seasons, tightly controlled collection, transportation, storage, and sample preparation procedures, and use of novel, precise, and widely accepted analytical methods for quantifying the nutrients present in samples. Pooling of a large number of eggs within each seasonal batch enabled us to provide measures that more accurately reflect the average nutrient content during each collection timepoint, at a reasonable cost. However, pooling masks the nutrient variability between eggs in a seasonal batch because the standard deviation reflects that of laboratory precision. Thus, the differences between the two seasonal batches of eggs may reflect both laboratory precision and natural variation in nutrient content. Analysis of eggs based on seasonality enabled us to better illustrate the extent of variation among individual nutrients, an important aspect in human nutrition research that is universally under‐recognized within food composition databases. Our research offers data that have the potential to supplement food composition databases. The study is also limited by the collection of samples in a single year. Other limitations include a lack of control over farming practices, even though this could be viewed as representative of what could be expected outside of our clinical trial in a real‐world setting. In particular, the COVID‐19 pandemic disrupted many feed supply chains, and these often‐undocumented changes could potentially influence the nutrient composition of the eggs.

## 5 CONCLUSION

The nutrient composition of eggs produced in the highlands of central Guatemala differed based on seasonality; however, only vitamins A and E, folate, choline, and calcium fluctuated at clinically significant levels relative to the AI/RDA for infants 7 to 12 months. Further multi‐year sampling is needed to examine how seasonal variation affects the nutrient composition of eggs. These data may be used to supplement existing national and regional food composition databases.

## AUTHOR CONTRIBUTIONS

**Taylor Wallace:** Conceptualization (lead); data curation (supporting); formal analysis (lead); funding acquisition (lead); investigation (lead); methodology (lead); project administration (lead); resources (supporting); software (supporting); supervision (lead); validation (lead); visualization (lead); writing – original draft (lead); writing – review and editing (lead). **Gabriela Montenegro‐Bethancourt:** Conceptualization (equal); data curation (supporting); formal analysis (equal); investigation (equal); methodology (equal); project administration (equal); resources (supporting); software (supporting); supervision (equal); validation (equal); visualization (equal); writing – original draft (equal); writing – review and editing (equal). **Peter Rohloff:** Conceptualization (equal); data curation (supporting); formal analysis (supporting); investigation (equal); methodology (equal); project administration (equal); resources (supporting); software (supporting); supervision (equal); validation (equal); visualization (equal); writing – original draft (equal); writing – review and editing (equal). **Elizabeth Yakes Jimenez:** Conceptualization (equal); data curation (supporting); formal analysis (supporting); investigation (equal); methodology (equal); project administration (equal); resources (supporting); software (supporting); supervision (equal); validation (equal); visualization (equal); writing – original draft (equal); writing – review and editing (equal). **Gabriela V. Proaño:** Conceptualization (equal); data curation (supporting); formal analysis (supporting); investigation (equal); methodology (equal); project administration (equal); resources (supporting); software (supporting); supervision (equal); validation (equal); visualization (equal); writing – original draft (equal); writing – review and editing (equal). **George P. McCabe:** Conceptualization (equal); data curation (supporting); formal analysis (supporting); investigation (equal); methodology (equal); project administration (equal); resources (supporting); software (supporting); supervision (equal); validation (equal); visualization (equal); writing – original draft (equal); writing – review and editing (equal). **Alison Steiber:** Conceptualization (equal); data curation (supporting); formal analysis (supporting); investigation (equal); methodology (equal); project administration (equal); software (supporting); supervision (supporting); validation (equal); visualization (equal); writing – original draft (equal); writing – review and editing (equal). **Andrew Ruosch:** Conceptualization (supporting); data curation (equal); formal analysis (equal); investigation (equal); methodology (equal); project administration (equal); resources (equal); software (equal); supervision (supporting); validation (supporting); visualization (supporting); writing – original draft (supporting); writing – review and editing (supporting). **Ian Laessig:** Conceptualization (supporting); data curation (equal); formal analysis (equal); investigation (equal); methodology (equal); project administration (equal); resources (equal); software (equal); supervision (supporting); validation (supporting); visualization (supporting); writing – original draft (supporting); writing – review and editing (supporting). **Edward Ladwig:** Conceptualization (supporting); data curation (equal); formal analysis (equal); investigation (equal); methodology (equal); project administration (equal); resources (equal); software (equal); supervision (supporting); validation (supporting); visualization (supporting); writing – original draft (supporting); writing – review and editing (supporting). **Hong You:** Conceptualization (supporting); data curation (equal); formal analysis (equal); funding acquisition (equal); investigation (supporting); methodology (supporting); project administration (equal); resources (equal); software (equal); supervision (equal); validation (supporting); visualization (supporting); writing – original draft (supporting); writing – review and editing (supporting).

## FUNDING INFORMATION

The Saqmolo’ Project was supported by the Academy of Nutrition and Dietetics Foundation via an investigator‐initiated research grant from the Egg Nutrition Center. The authors and sponsor strictly adhere to the American Society for Nutrition's guiding principles for private funding for food science and nutrition research (Rowe et al., 2009). The funder had no role in the design of this study; nor will the funder have influence over the execution of the study, data analysis, or reporting of results. The team is contractually obligated to publish the results of the study regardless of the findings. In‐kind nutrient analyses for this study were provided by Eurofins US Food.

## CONFLICT OF INTEREST STATEMENT

TCW, PR, GM‐B, GPM, and AS receive salary support through the investigator‐initiated grant provided to the Academy of Nutrition and Dietetics Foundation by the Egg Nutrition Center. GM‐B is a research fellow funded by the Academy of Nutrition and Dietetics Foundation through the investigator‐initiated grant from the Egg Nutrition Center. TCW has received other research grants and scientific consulting fees from the Egg Nutrition Center. AR, IL, EL, and HY are employees of Eurofins US Food. EYJ and GVP have no conflicts of interest to report.

## TRIAL REGISTRATION

ClinicalTrials.gov identifier NCT04316221.

## Data Availability

The data that support the findings of this study are available from the corresponding author upon reasonable request. Data were provided to reviewers during peer review of the manuscript.
